# Assessment of Body Composition as an Indicator of Early Peripheral Parenteral Nutrition Therapy in Patients Undergoing Colorectal Cancer Surgery in an Enhanced Recovery Program

**DOI:** 10.3390/nu13093245

**Published:** 2021-09-18

**Authors:** Francisco López-Rodríguez-Arias, Luis Sánchez-Guillén, Cristina Lillo-García, Verónica Aranaz-Ostáriz, M José Alcaide, Álvaro Soler-Silva, Leticia Soriano-Irigaray, Xavier Barber, Antonio Arroyo

**Affiliations:** 1 Colorectal Unit, Department of General Surgery, Elche University Hospital, Miguel Hernandez University, 03202 Elche, Spain; franloarias@hotmail.com (F.L.-R.-A.); cristina.lillo93@gmail.com (C.L.-G.); veronica.aranaz@gmail.com (V.A.-O.); mjose_alcaide@hotmail.com (M.J.A.); soler.medi@gmail.com (Á.S.-S.); arroyocir@hotmail.com (A.A.); 2 Department of Pharmacy, Elche University Hospital-FISABIO, 03202 Elche, Spain; leticiasorianoirigaray@gmail.com; 3 Center for Operations Research, Miguel Hernandez University, 03202 Elche, Spain; xbarber@umh.es

**Keywords:** peripheral parenteral nutrition, colorectal cancer, body composition, postoperative complications, ERAS

## Abstract

Background: A poor body composition (BC) has been identified as a risk factor for patients with colorectal cancer (CRC). This study was performed to assess the effect of early peripheral parenteral nutrition (PPN) on BC in patients undergoing CCR surgery within an enhanced recovery program. Methods: Patients with normal nutritional status were prospectively included between October 2016 and September 2019, randomized into two groups (PPN with periOlimel N4-E versus conventional fluid therapy) and subsequently classified according to their preoperative CT scan into high- or low-risk BC groups. Postoperative complications and length of hospital stay (LOS) were assessed. Results: Of the 156 patients analyzed, 88 patients (56.4%) were classified as having high-risk BC according to CT measurements. PPN led to a 15.4% reduction in postoperative complications in high-risk vs. 1.7% in low-risk BC patients. In the multivariate analysis, high-risk BC was related to an OR (95% CI) of 2 (*p* = 0.044) of presenting complications and of 1.9 (*p* = 0.066) for major complications, and was associated with an increase in LOS of 3.6 days (*p* = 0.039). Conclusions: The measurement of patients’ BC can allow for the identification of target patients where PPN has been proven to be an effective tool to improve postoperative outcomes.

## 1. Introduction

In the past, body mass index (BMI) has been traditionally used as an indicator of malnutrition and prognosis. However, in recent years, the number of obese patients has increased, pointing to limitations in BMI as a nutritional risk and prognosis indicator [1].

Body composition, measured by computed tomography (CT), is easily identifiable next to the L3 vertebra level, and allows for calculation of the skeletal muscle index (SMI). It has been used in recent studies to evaluate the relationship between skeletal muscle mass and postoperative outcomes. Low SMI has been identified as a predictor of a poor outcome for patients with operable CRC in terms of both short- and long-term clinical outcomes [2,3,4]. The importance of body composition has been reflected by it being included in the diagnosis of malnutrition by the GLIM criteria [5].

Colorectal cancer (CRC) is the third most common cancer and the fourth leading cause of cancer-related deaths worldwide. Surgery is one of the fundamental curative treatments for CRC [6,7]. However, despite advances in perioperative medicine that improve survival, such as multimodal or fast-track rehabilitation programs based on Enhanced Recovery After Surgery (ERAS) guidelines, it is still associated with high rates of mortality and postoperative complications. One of the main factors involved in ERAS programs is the nutritional status assessment of patients with cancer [8].

Early postoperative nutrition after lower gastrointestinal surgery may lead to reduced postoperative complications, but additional studies are needed [9]. A growing number of studies have evaluated the effect of nutritional support on clinical and nutritional outcomes. However, how the different types of nutritional interventions impact nutritional status in different settings is still being debated.

PeriOlimel N4-E is a peripheral parenteral nutrition comprised of lipids, glucose and amino acids that can be used as a substitute for or as a complement to enteral nutrition. It is high in oleic acid, which could better preserve the patient’s immune response, decrease oxidative stress, and reduce inflammation. This can improve the inflammatory response and reduce tissue degradation, preventing significant weight loss, alterations in body composition, and decreased functional capacity [10].

There are no previous studies that have analyzed the effect of perioperative supplementation with peripheral parenteral nutrition (PPN) during the first postoperative days (when nutritional input is not complete) on postoperative outcomes in CRC patients with a poor body composition.

The aim of this study was to evaluate whether early postoperative supplementation with periOlimel N4-E versus conventional fluid therapy (FT) improves postoperative outcomes and LOS according to the body composition measured by the Skeletal Muscle Index in patients with CRC within an ERAS program.

## 2. Materials and Methods

### 2.1. Study Design

The present study is a sub-analysis of a procedure-targeted cohort of patients obtained from a randomized clinical trial of superiority conducted from October 2016 to September 2019. It compares the influence of PPN with PeriOlimel N4-E vs. conventional FT depending on body composition according to the SMI on postoperative complications of colorectal surgery patients [11].

The inclusion criteria were as follows: individuals aged ≥18 years and a diagnosis of colorectal cancer with preoperative staging T1-T3NxM0.

The exclusion criteria were patients at severe nutritional risk via one of the ESPEN guidelines criteria (weight loss >10–15% within 6 months, BMI < 18.5 kg/m^2^, SGA grade C or NRS > 5, and preoperative serum albumin < 30 g/L without evidence of liver or kidney dysfunction) [12,13], intraoperative diagnosis of carcinomatosis, metastasis, and locally advanced (T4) or unresectable tumors. Other exclusion criteria included the need for emergency surgery, an American Society of Anaesthesiologists (ASA) physical status IV, renal failure defined via hemodialysis, hepatic failure, allergy or sensitivity to egg or soy protein, severe bleeding disorder, congenital abnormality of amino acid metabolism hyperlipidemia, not accepting or not being able to comply with the ERAS protocol, or the absence of a CT scan one month prior to surgery.

The study was approved by the Hospital General Universitario de Elche Ethics Committee. The research was conducted in accordance with the Helsinki Declaration and local legislation. All patients were informed about the study, invited to participate and signed an informed consent statement before starting the study. This study has been registered in the NCT register as NCT03606863.

### 2.2. Randomization

Patients were assigned (1:1) with double-blind randomization to receive traditional fluid therapy (control group) versus peripheral nutrition with Peri-Olimel N4-E (PPN group) using online randomization software.

### 2.3. Study Protocol

All patients underwent perioperative management following the current indications of the ERAS protocols [8]. Patients were preoperatively prepared at home with a low-fiber diet three days before surgery and admitted to the hospital the day before the surgery.

Antibiotic prophylaxis was administered following the policy of our center; in addition, patients received drinks rich in carbohydrates with dextromaltose a day before the surgery and on the morning of the surgery.

In the intervention group, PPN with Peri-Olimel N4-E was started one day prior to colorectal resection and continued for 3 days postoperatively. In the control group, standard FT was administered postoperatively and removed when the patient began to tolerate oral feeds.

All patients underwent surgery performed by surgeons from the coloproctology unit, giving priority to laparoscopic surgery.

Intraoperative goal-directed fluids, hypothermia and drainage tubes were avoided, and epidural anesthesia (ropivacaine 0.2%) was used only in open procedures. Nasogastric tubes were not used, opioid-free pain control and prophylactic medication for nausea and vomiting were used, and oral fluids were administered early.

Early mobilization and tolerance were practiced postoperatively in both groups.

### 2.4. Body Composition Protocol

**Figure 1:**
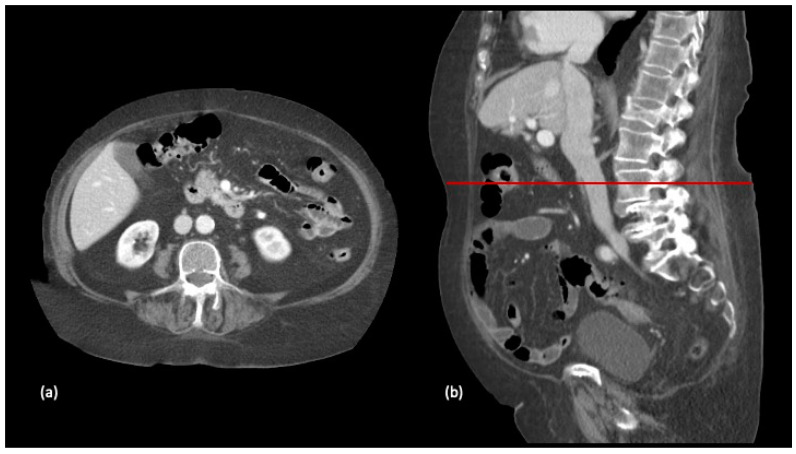
Selection of axial (**a**) and sagittal (**b**) slices at the level of the third lumbar vertebra in a preparatory computed tomography study.

**Figure 2:**
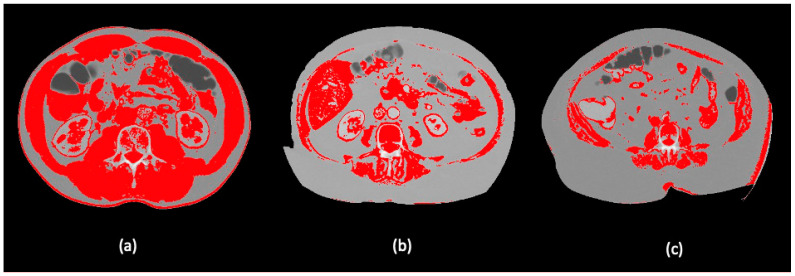
Visualization of different body composition models applying a skeletal muscle threshold of -29-150 HU in the ImageJ software: (**a**) high SMI; (**b**) low SMI and (**c**) morbid obesity with high SMI in Dolan´s classification.

The participants were classified according to their SMI assessed from images at the L3 vertebra level on the preoperative CT scan, performed one month prior to hospital admission (Figure 1 and Figure 2).

Images were analyzed using the NIH image software ImageJ (https://imagej.nih.gov.ij/ (accessed on 1 April 2021)), employing a skeletal muscle threshold of -29-150 HU and following the steps proposed in the study of Gomez-Perez S, et al. [14]

Measurements were carried out by two researchers who had previously performed a joint measurement of 20 patients showing an intraclass correlation coefficient of 0.97.

The SMI threshold values obtained were similar to those of Dolan et al. [15]. High SMI was defined for males as SMI ≥ 45 cm^2^m^2^ if BMI < 25 kg/m^2^ and SMI ≥ 53 cm^2^m^2^ if BMI ≥ 25 kg/m^2^, and for females as SMI ≥ 39 cm^2^m^2^ if BMI < 25 kg/m^2^ and SMI ≥ 41 cm^2^m^2^ if BMI ≥ 25 kg/m^2^; and low SMI was defined for males as SMI < 45 cm^2^m^2^ if BMI < 25 kg/m^2^ and SMI < 53 cm^2^m^2^ if BMI ≥ 25 kg/m^2^, and for females as SMI < 39 cm^2^m^2^ if BMI < 25 kg/m^2^ and SMI <41 cm^2^m^2^ if BMI ≥ 25 kg/m^2^.

### 2.5. Data Collection

A confidential database was prepared for the collection of data.

The following variables were analyzed: demographic data (age, sex), comorbidities (ASA score, oral anticoagulants, smoking habit, high blood pressure and diabetes), surgical details (surgical approach, tumor location, type of anastomosis, and perioperative transfusions) and characteristics of the disease (cancer vs. benign and pathological details with the TNM system).

Complications and mortality were evaluated 90 days after surgery using the Clavien–Dindo classification and divided into minor (classified as Clavien–Dindo I–II), which included low-risk events such as surgical wound infection or postoperative ileus, and major (Clavien–Dindo III–IV) [16].

Compliance with ERAS was determined and recorded in the database (incidence of postoperative nausea or vomiting, onset of tolerance, type of tolerated diet and the onset of ambulation).

### 2.6. Data Analysis and Simple Size Calculation

Categorical variables were reported as the number of patients and the percentage, and analyzed using χ2 tests. *p* < 0.05 was considered to indicate statistical significance.

Two types of multivariate analyses were performed: logistic regression with the independent variables and multiple linear regression with risk factors related to the length of hospital stay.

We performed all analyses using R software and the rpart package [17,18].

Sample size calculation was performed to compare the incidence of postoperative complications between PPN and conventional FT, which was expected to be 0.35 for the control group and 0.17 for the intervention group. We found that 170 patients were required, 85 patients per group, with a confidence level of 95% (alpha = 0.05) and a power of 80% (beta = 0.2) in a bilateral contrast, to detect as statistically significant the difference between the two proportions, assuming 10% losses.

## 3. Results

A total of 170 patients were consecutively enrolled in the clinical trial, 14 of whom were excluded because they did not meet the previously established criteria. A total of 52.6% of the patients were assigned to the PPN group (82 patients), and 47.4% were assigned to the FT group (74 patients). Their average age was 69.5 years (71.1 years in the PPN group vs. 67.7 years in the FT group); 61.5% of the patients were male, and their average BMI was 27.9 kg/m^2^ (27.8 kg/m^2^ in the PPN group vs. 28.1 kg/m^2^ in the FT group). Complications occurred in 38.5% of the patients who underwent surgery, of which 40% were major (Clavien–Dindo III–V) and 60% were minor (Clavien–Dindo I–II). In addition, the median LOS was 6 days, and 35.9% of the patients had an LOS greater than or equal to 7 days.

Table 1 shows the relationship between the clinical and pathological characteristics and the postoperative outcomes after classifying patients into high or low SMI according to the classification of Dolan et al. [10]. In our study, 50% of the patients presented with high SMI and 50% with low SMI. Age showed a statistically significant relationship (*p* < 0.001), so the percentage of high SMI was greater in younger patients (70.9% in those <65 years), while low SMI was more frequent in older patients (73.8% of patients >75 years old).

Patients with ASA scores I–II were classified mainly as having a high SMI (63.8% high SMI vs. 36.2% low SMI), and those with ASA scores III–IV were classified as having a low SMI (29% high SMI vs. 71% low SMI) (*p* < 0.001). There were no differences in sex or patient distribution in the PPN or FT group. Regarding BMI, we found statistically significant differences (*p* = 0.021), highlighting that in the BMI range of 25 to 35, patients were preferentially classified as having a low SMI (43% high SMI vs. 57% low SMI), while in the BMI range of >35, they were more commonly classified as having a high SMI (78.6% high SMI vs. 21.4% low SMI). Finally, complications were more frequent in the low SMI group than in the high SMI group, but the difference was not statistically significant.

**Figure 3:**
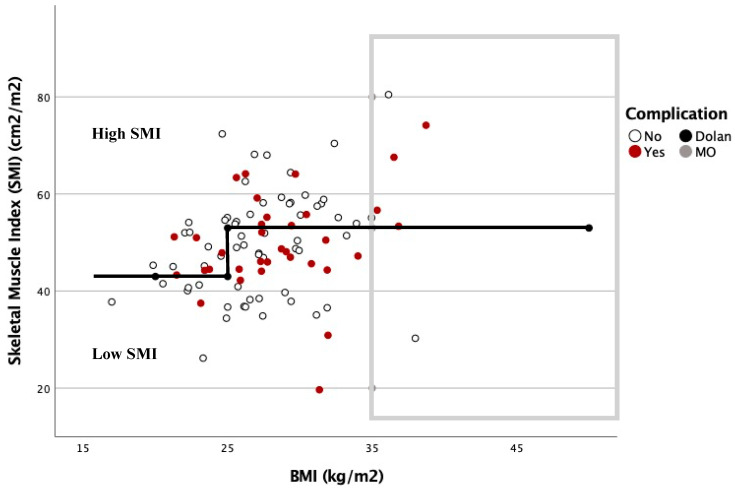
Dispersion diagram correlating complications between BMI and SMI in men.

**Figure 4:**
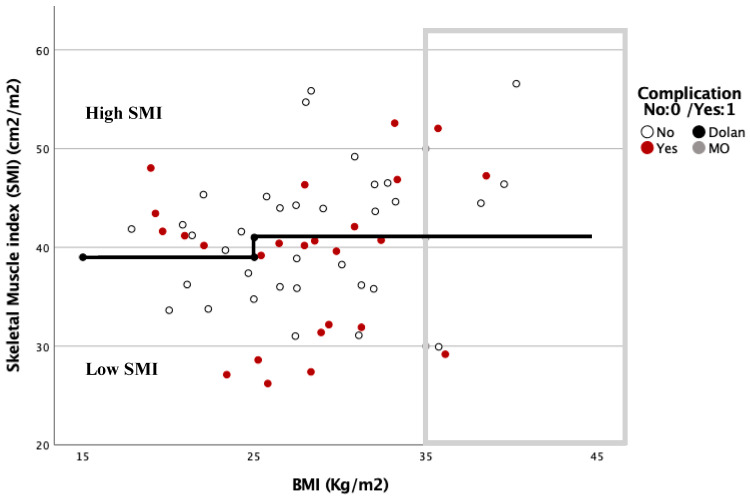
Dispersion diagram correlating complications between BMI and SMI in women.

The relationship between the complications based on the SMI and BMI is shown in Figure 3 and Figure 4. In these graphs, complications are more frequent under the Dolan classification line for high or low SMI. However, with a BMI ≥ 35 kg/m^2^, 78.6% of the patients were located above this line, and presented with 57.1% postoperative complications.

Therefore, we propose a new classification based on Dolan´s, called the Body Composition Elche (BCE) classification, for further analysis and classification of the patients’ body composition (BC) into low- or high-risk groups. Low-risk BC is defined as a high SMI of Dolan´s classification, excluding patients with BMI ≥ 35 kg/m^2^, and high-risk BC is defined as a low SMI plus patients with BMI ≥ 35 kg/m^2^.

Table 2 shows the results obtained when classifying patients via the BCE classification into two categories: low-risk BC and high-risk BC. In total, 68 patients (43.6%) were classified as low-risk, and 88 patients (56.4%) were classified as high-risk BC.

Age, ASA score and BMI continued to have a statistically significant relationship with our classification. There were no differences in sex or patient distribution in the PPN or FT group. In our classification, patients at low risk had 29.4% complications compared to 45.5% for those with high-risk BC (*p* = 0.041). Major complications and postoperative ileus were also higher in these patients (8.8% vs. 20.5%, *p* = 0.046; and 5.9% vs. 22.7%, *p* = 0.004).

Table 3 shows the relationship between PPN and complications according to the body composition of the patient. A 15.4% reduction in the percentage of complications was observed in patients at high risk who were given PPN compared to those who only received FT. In the low-risk group, the addition of PPN only resulted in a 1.7% reduction in complications compared to FT (*p* = 0.041).

With respect to major complications, a reduction of 7.4% was observed with PPN in patients classified as high-risk versus a 6% reduction in patients classified as low-risk BC (*p* = 0.046).

Of the 14 patients with a BMI ≥35, 8 patients received PPN (37.5% complications and 12.5% major complications), and 6 patients received conventional FT (66.7% complications and 33.3% major complications).

Logistic regression was performed with uni- and multivariate analyses to assess the risk of presenting with complications and major complications depending on the body classification of the patient with high- or low-risk BC, and whether they received PPN or FT.

In the univariate analysis, high-risk BC was associated with an OR (95% CI) of 2 (1.02–3.91, *p* = 0.060) for presenting any complication, and of 2.66 (0.99–7.12, *p* = 0.763) for major complications, while PPN was associated with an OR (95% CI) of 0.68 (0.36–1.30, *p* = 0.3166) for presenting any complication and of 0.6 (0.25–1.44, *p* = 0.347) for major complications.

In the multivariate analysis, high-risk BC was associated with an OR (95% CI) of 2 (1.03–3.98, *p* = 0.044) for presenting any complication and of 1.9 (0.97–3.8, *p* = 0.066) for major complications, and PPN was associated with an OR of 0.67 (0.34–1.29, *p* = 0.228) for presenting any complication and of 0.67 (0.34–1.29, *p* = 0.232) for major complications.

Table 4 shows our multiple linear regression analysis, which identifies the main risk factors related to LOS. The following independent variables were included in the analysis: age >65 years, female gender, administration of PPN, ASA III, high-risk BC, BMI 25–35 and BMI > 35. Patients had an LOS of 6.06 days (95% CI) (*p* = 0.003), which was modified by the effect of the variables studied. Body composition was the variable that had the most significant effect on the LOS, so patients with high-risk BC showed an increase of 3.6 days (95% CI) (*p* = 0.039).

## 4. Discussion

To the best of our knowledge, this is the first study to evaluate the effect of perioperative supplementation with PPN on postoperative outcomes in CRC patients operated on within an ERAS program according to their BC risk, as measured by the SMI. Multivariate analysis showed that we could determine the risk of postoperative complications and LOS based on patient body composition, and PPN seems to reduce postoperative complications.

Complications following CRC surgery increase the LOS and cost, and decrease the long-term survival of patients [19,20]. The POWER study analyzed the incidence of complications in 2084 patients operated on for RCC according to their adherence to the ERAS protocols [21]. Despite the fact that the average age of our patients was older (69.5 years vs. 61.7 years), our rate of complications was similar to that reported in centers with a high level of compliance with ERAS programs (38.5% vs. 40.72), although our rate of moderate to severe complications (Clavien–Dindo III–IV) was lower (15.4 vs. 25.2). The rate of paralytic ileus here (15.4%) was between the values collected in centers with high or low ERAS compliance (13.34% to 17.31%).

To try to prevent postoperative complications and to mitigate their consequences, in recent years, some studies have been published that have tried to identify perioperative risk factors that are able to predict the occurrence of postoperative complications [22,23]. Due to this, a promising area of investigation on BC has emerged. Currently, we know that low muscle mass acts as an independent risk factor with a negative impact on short- and long-term clinical outcomes.

The study by Abbass et al. [24] analyzed the results of 1002 patients with CRC who were classified according to their SMI by Dolan´s classification [15]. In our study, we found that the percentages of patients classified as having a high SMI or low SMI were similar to the data obtained from the analysis of Abbass et al. Similarly, age, ASA score and BMI presented a statistically significant relationship with this classification. The difference in complications between the high-SMI vs. low-SMI groups was greater (32.1–44.9% vs. 36.9 vs. 40.5%) in our study, as was the difference in major complications (11.5–19.2% vs. 7.6–11.3%). We did not obtain statistically significant differences in our study with this classification, and Abbass et al. only obtained significance for major complications.

There is an association between obesity and the development of colorectal cancer [25]. In addition, obesity increases the risk of complications after CRC surgery. Several studies report an OR of 2.1 for complications, among which infection of the surgical site stands out [26,27]. However, there are doubts about establishing the exact cut-off point for the BMI, at which the increase in complications begins to become exponential. Several studies have reported that there is a certain paradox in the relationship between BMI survival and CRC, observing that patients with overweight or obesity grade 1 had better survival than patients with extreme values of either overweight or underweight [1,28].

Body composition studies analyzing the SMI or Psoas Muscle Index (PMI) are helping us to understand the mechanisms underlying these results. However, as we can see in our study and in what is described in the literature, SMI is influenced by BMI [24]. There are different cut-off points for SMI that try to identify where the limit of sarcopenia or surgical risk is [15,23]. Caan et al. increased the SMI limit value from a BMI ≥ 30 to 54.3 in males and 46.6 in females [28]; however, given the intrinsic surgical risk that exists in patients with obesity, the cut-off point that identifies the risk will follow a function with exponential behavior, in such a way that there will be a BMI threshold after which, regardless of SMI, the patient must be classified as a high-risk patient.

For all of these reasons, we proposed the BCE classification in an attempt to approximate this exponential function between BMI and SMI, in order to better classify patients according to their surgical risk.

The restaging of patients according to the BCE classification meant a transfer of 6.4% of patients classified as low surgical risk (high SMI) to the high-risk group. This increased the difference between the groups as regards general complications (29.4–45.4%, *p* = 0.041), major complications (8.8–20.5%, *p* = 0.046) and paralytic ileus (5.9–22.7%, *p* = 0.004).

Multivariate analysis showed that high-risk BC patients had a doubled risk of developing complications, and this was also one of the factors that most affected the increase in LOS. Although it did not reach statistical significance, PPN was the only parameter analyzed that reduced the LOS by −0.56 days (*p* = 0.7).

Our classification of body composition has been shown to improve the prediction of complications, but additional studies with very large samples will be needed to construct the different functions for men and women that identify the optimal BMI/SMI cut-off point to locate patients at high surgical risk who may benefit from perioperative treatments, such as PPN. These functions will be more or less restrictive, and the aggressiveness of the surgery will not delimit the extent of sarcopenia, only the surgical risk.

There is evidence that nutritional intervention in undernourished patients improves postoperative outcomes, decreasing the incidence of complications [29]. In addition, protocolized early oral tolerance in ERAS programs has been shown to accelerate gastrointestinal recovery, decreasing postoperative complications and LOS [30,31].

However, there are no previous studies that have assessed the effects of PPN in well-nourished patients (according to the classic criteria of malnutrition) with the early initiation of tolerance by the oral route, and evaluated this effect according to the characteristics of the patient’s body composition. Our results suggest that patients classified as high-risk BC clearly benefited from perioperative treatment with PPN.

## 5. Conclusions

The analysis of the body composition of patients through the determination of SMI is a useful tool to identify patients at high surgical risk. In these patients, peripheral parenteral nutrition has been shown to be effective in improving the outcomes of surgery, and could contribute to reducing the length of hospital stay.

## Figures and Tables

**Table 1:** The relationship between the clinicopathological characteristics and the clinical outcomes stratified by SMI.

Characteristics	All, *n* (%) *n* = 156	High SMI, *n* (%) *n* = 78 (50)	Low SMI, *n* (%) *n* = 78 (50)	*p*-Value
Age				<0.001
<65	55 (35.3)	39 (50)	16 (20.5)	
65–75	40 (25.6)	23 (29.5)	17 (21.8)	
>75	61 (39.1)	16 (20.5)	45 (57.7)	
Sex				0.999
Male	96 (61.5)	48 (61.5)	48 (61.5)	
Female	60 (38.5)	30 (38,5)	30 (38.5)	
ASA score				<0.001
I–II	94 (60.3)	60 (76.9)	34 (43.6)	
III	62 (39.7)	18 (23.1)	44 (56.4)	
BMI				0.021
<25	42 (26.9)	24 (30.8)	18 (23.1)	
25–35	100 (64.1)	43 (55.1)	57 (73.1)	
>35	14 (9)	11 (14.1)	3 (3.8)	
Group				0.999
PPN	82 (52.6)	41 (52.6)	41 (52.6)	
Control	74 (47.4)	37 (47.4)	37 (47.4)	
Complications				0.1
Yes	60 (38.5)	25 (32.1)	35 (44.9)	
No	96 (61.5)	53 (67.9)	43 (55.1)	
Minor				0.447
Yes	36 (23.1)	16 (20.5)	20 (25.6)	
No	120 (76.9)	62 (79.5)	58 (74.4)	
Major				0.183
Yes	24 (15.4)	9 (11.5)	15 (19.2)	
No	132 (84.6)	69 (88.5)	63 (80.8)	
Postoperative Ileus				0.076
Yes	24 (15.4)	8 (0,1)	16 (20.5)	
No	132 (84.6)	70 (0,9)	62 (79.5)	
Length of hospital stay				0.317
≤7 days	100 (64.1)	53 (67.9)	47 (60.3)	
>7 days	56 (35.9)	25 (32.1)	31 (39.7)	
Sitting in a chair (POD1)				0.363
Yes	115 (73.7)	60 (76.9)	55 (70.5)	
No	41 (26.3)	18 (23.1)	23 (29.5)	
Oral Tolerance (POD1)				0.057
Yes	129 (82.7)	69 (88.5)	60 (76.9)	
No	27 (17.3)	9 (11.5)	18 (23.1)	

SMI: Skeletal Muscular Index, ASA: American Society of Anesthesiologists, BMI: Body Mass Index, PPN: Parenteral Peripheral Nutrition, POD1: Post-operative day 1 of surgery. Chi-Square was used for low and high-risk body composition (bilateral significance).

**Table 2:** The relationship between clinicopathological characteristics and clinical outcomes stratified by low- or high-risk body composition in the BCE classification.

Characteristics	All, *n* (%) *n* = 156	Low Risk BC, *n* (%) *n* = 68 (43.6)	High Risk BC, *n* (%) *n* = 88 (56.4)	*p*-Value
Age				<0.001
<65	55 (35.3)	32 (47.1)	23 (26.1)	
65–75	40 (25.6)	22 (32.4)	18 (20.5)	
>75	61 (39.1)	14 (20.6)	47 (53.4)	
Sex				0.475
Male	96 (61.5)	44 (64.7)	52 (59.1)	
Female	60 (38.5)	24 (35.3)	36 (40.9)	
ASA				<0.001
I–II	94 (60.3)	53 (77.9)	41 (46.6)	
III–V	62 (39.7)	15 (22.1)	47 (53.4)	
BMI				<0.001
<25	42 (26.9)	25 (36.8)	27 (30.7)	
25–35	100 (64.1)	43 (63.2)	57 (64.8)	
>35	14 (9)	0	14 (15.9)	
Group				0.81
PPN	82 (52.6)	35 (0.5)	41 (46.6)	
Control	74 (47.4)	33 (0.5)	47 (53.4)	
Complications				0.041
Yes	60 (38.5)	20 (29.4)	40 (45.5)	
No	96 (61.5)	48 (70.6)	48 (54.5)	
Minor				0.517
Yes	36 (23.1)	14 (20.6)	22 (25)	
No	120 (76.9)	54 (79.4)	66 (75)	
Major *n*, (SD)				0.046
Yes	24 (15.4)	6 (8.8)	18 (20.5)	
No	132 (84.6)	62 (91.2)	70 (79.5)	
Postoperative Ileus				0.004
Yes	24 (15.4)	4 (5.9)	20 (22.7)	
No	132 (84.6)	64 (94.1)	68 (77.3)	
Length of hospital stay				0.069
≤7 days	100 (64.1)	49 (72.1)	51 (58)	
>7 days	56 (35.9)	19 (27.9)	37 (42)	
Sitting in a chair (POD1)				0.156
Yes	115 (73.7)	54 (79.4)	61 (69.3)	
No	41 (26.3)	14 (20.6)	27 (30.7)	
Oral Tolerance (POD1)				0.042
Yes	129 (82.7)	61 (89.7)	68 (77.3)	
No	27 (17.3)	7 (10.3)	20 (22.7)	

BC: body composition; ASA: American Society of Anesthesiologists; BMI: Body Mass Index, PPN: parenteral peripheral nutrition; POD1: Post-operative day 1 of surgery. Chi-Square was used for low- and high-risk body composition (bilateral significance).

**Table 3:** Relationship of PPN and body composition with complications.

All *n* = 156	Low-Risk BC, *n* (%) *n* = 68 (43.6%)		High-Risk BC, *n* (%) *n* = 88 (56.4%)		*p*-Value
Group *n*, (%)	PPN *n* = 35 (51.5)	Control *n* = 33 (48.5)	PPN *n* = 47 (53.4)	Control *n* = 41 (46.6)	
Complications *n*, (%)	10 (28.57)	10 (30.30)	18 (38.3)	22 (53.66)	0.041
Minor *n*, (%)	8 (22.86)	6 (18.18)	10 (21.28)	12 (29.27)	0.517
Major *n*, (%)	2 (6.06)	4 (12.12)	8 (17.02)	10 (24.39)	0.046

BC: body composition, PPN: parenteral peripheral nutrition. Chi-square was used for low- and high-risk body composition (bilateral significance).

**Table 4:** Multiple linear regression with risk factors related to the LOS.

Independent Variables	β (95%CI)	*p*-Value
β_0_	6.06 (2.07,10.06)	0.003
β_1_ > 65 years	2.22 (−1.7,6.13)	0.269
β_2_ Female gender	0.28 (−2.72,3.28)	0.855
β_3_ PPN	−0.56 (−3.49,2.36)	0.706
β_4_ ASA III	0.33 (−3.14,3.8)	0.853
β_5_ High risk BC	3.6 (0.21,7)	0.039
β_6_ BMI 25–35	0.71 (−2.65,4.08)	0.678
β_7_ BMI >35	1.36 (−4.76,7.48)	0.663
Lenght hospital stays = β_0_ + β_1_ ≥ 65 years+⋯+ β_7_ BMI ≥ 35		

PPN: Peripheral parenteral nutrition; ASA: American Society of Anesthesiologists; BC: body composition; BMI: body mass index.

## Data Availability

The data presented in this study are available on request from the corresponding author. The data are not publicly available due to privacy restrictions.

## Abbreviations

BC	Body composition
BCE Classification	Body Composition Elche Classification
BMI	Body Mass Index
FT	Fluid therapy
PPN	Peripheral parenteral nutrition
SMI	Skeletal muscle index
